# A knowledge translation collaborative to improve the use of therapeutic hypothermia in post-cardiac arrest patients: protocol for a stepped wedge randomized trial

**DOI:** 10.1186/1748-5908-6-4

**Published:** 2011-01-14

**Authors:** Katie N Dainty, Damon C Scales, Steve C Brooks, Dale M Needham, Paul Dorian, Niall Ferguson, Gordon Rubenfeld, Randy Wax, Merrick Zwarenstein, Kevin Thorpe, Laurie J Morrison

**Affiliations:** 1 RESCU Research Program, Keenan Research Centre, Li Ka Shing Knowledge Institute, St. Michael's Hospital Toronto, Canada; 2 Department of Critical Care Medicine, Sunnybrook Health Sciences Centre, Institute for Clinical Evaluative Sciences, Interdepartmental Division of Critical Care, University of Toronto, Toronto, Canada; 3 Division of Pulmonary and Critical Care Medicine, and Department of Physical Medicine and Rehabilitation, School of Medicine, Johns Hopkins University, Baltimore USA; 4 Division of Cardiology, St. Michael's Hospital, University of Toronto, Toronto, Canada; 5 Department of Medicine, Division of Respirology, University Health Network and Mount Sinai Hospital, Toronto, Ontario, Canada; 6 Interdepartmental Division of Critical Care Medicine, University of Toronto, Toronto, Canada; 7 Department of Emergency Medicine and Critical Care, Lakeridge Health Corporation, Oshawa, Ontario, Canada; 8 Interdepartmental Division of Critical Care, University of Toronto, Toronto, Canada; 9 Sunnybrook Research Institute, Sunnybrook Health Sciences Centre; Department of Health Policy, Management and Evaluation, University of Toronto, Toronto, Canada; 10 Keenan Research Centre, Li Ka Shing Knowledge Institute, St. Michael's Hospital Toronto, Canada; 11 Faculty of Medicine, Division of Emergency Medicine, University of Toronto, Toronto Canada

## Abstract

**Background:**

Advances in resuscitation science have dramatically improved survival rates following cardiac arrest. However, about 60% of adults that regain spontaneous circulation die before leaving the hospital. Recently it has been shown that inducing hypothermia in cardiac arrest survivors immediately following their arrival in hospital can dramatically improve both overall survival and neurological outcomes. Despite the strong evidence for its efficacy and the apparent simplicity of this intervention, recent surveys show that therapeutic hypothermia is delivered inconsistently, incompletely, and often with delay.

**Methods and design:**

This study will evaluate a multi-faceted knowledge translation strategy designed to increase the utilization rate of induced hypothermia in survivors of cardiac arrest across a network of 37 hospitals in Southwestern Ontario, Canada. The study is designed as a stepped wedge randomized trial lasting two years. Individual hospitals will be randomly assigned to four different wedges that will receive the active knowledge translation strategy according to a sequential rollout over a number of time periods. By the end of the study, all hospitals will have received the intervention. The primary aim is to measure the effectiveness of a multifaceted knowledge translation plan involving education, reminders, and audit-feedback for improving the use of induced hypothermia in survivors of cardiac arrest presenting to the emergency department. The primary outcome is the proportion of eligible OHCA patients that are cooled to a body temperature of 32 to 34°C within six hours of arrival in the hospital. Secondary outcomes will include process of care measures and clinical outcomes.

**Discussion:**

Inducing hypothermia in cardiac arrest survivors immediately following their arrival to hospital has been shown to dramatically improve both overall survival and neurological outcomes. However, this lifesaving treatment is frequently not applied in practice. If this trial is positive, our results will have broad implications by showing that a knowledge translation strategy shared across a collaborative network of hospitals can increase the number of patients that receive this lifesaving intervention in a timely manner.

**Trial Registration:**

ClinicalTrials.gov Trial Identifier: NCT00683683

## Background

**Figure 1:**
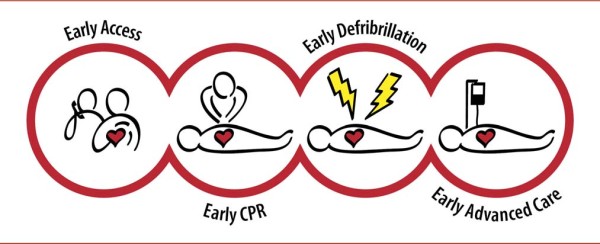
**Chain of survival for out-of-hospital cardiac arrest**.

Out of hospital cardiac arrest (OHCA) can be a devastating event. Only about one-third of patients regain pulses with resuscitation after OHCA, and less than half of patients admitted to hospital survive to hospital discharge [1]. Many of these survivors will have permanent neurological impairment caused by anoxic brain injury. Current advanced cardiac life-support (ACLS) algorithms for cardiac arrest have traditionally focused on early intensive resuscitation, and options to prevent anoxic brain injury have been mostly limited to supportive care (Figure 1).

Recently it has been shown that induced hypothermia applied immediately after hospital arrival can improve survival with good neurologic outcome and is now a recommended therapy for patients resuscitated from cardiac arrest [2]. This therapy involves cooling patients to 32 to 34°C for 12 to 24 hours following the return of spontaneous circulation. Although its mechanism is not completely understood, a reduction in core body temperature likely diminishes cellular injury and increases cerebral neuronal healing by reducing cerebral oxygen demand and intracranial pressure. Through these mechanisms, induced hypothermia is thought to attenuate post-ischemic hypo-perfusion, stabilize plasma membranes, and suppress the production and release of free radicals [3]. The evidence supporting therapeutic hypothermia includes one randomized control trial [4] with American Heart Association (AHA) Level 1 evidence; two smaller quasi-randomized studies [5,6] (AHA Level 2 evidence); three prospective, non-randomized studies [7-9] (AHA Level 3 to 5 evidence) and multiple animal model studies. All studies demonstrate that therapeutic hypothermia protects the brain from the late deleterious consequences of the hypoxic/ischemic injury post-arrest. A meta-analysis demonstrated more favourable neurologic recovery with therapeutic hypothermia (risk ratio 1.69, 95% confidence intervals 1.29 to 2.07) and found that the number needed to treat for neurologically intact survival was six patients [10].

### The Knowledge Gap

The AHA, International Liaison Committee on Resuscitation, the Canadian Association of Emergency Physicians, and other national and international agencies strongly recommend the rapid institution of therapeutic hypothermia in eligible patients following resuscitation from cardiac arrest [2]. However, observational research shows that therapeutic hypothermia is delivered inconsistently, incompletely, and often with delay. For example, in surveys of hospitals receiving resuscitated patients, only 26% of physicians [11,12] (USA and Canada) and 26% of hospitals [13] (United Kingdom) reported regularly instituting an induced hypothermia protocol. A recent Canadian survey of emergency and critical care physicians showed that most respondents had knowledge of induced hypothermia (99%) and considered it to be beneficial (91%), but only two-thirds (68%) had used it in clinical practice [14]. Reasons cited to explain this lack of adoption included lack of awareness of recommended practice (31%), perceptions of poor prognosis (25%), too much work required to cool (20%), and staffing shortages (20%). Another recent survey of Canadian emergency medicine physicians revealed that only about one-third of departments had a therapeutic hypothermia policy or protocol and that the presence of a policy or protocol strongly predicted the use of therapeutic hypothermia [15].These results suggest that strategies are required to increase the use of induced hypothermia for cardiac arrest survivors.

We hypothesize that two main factors contribute to the poor implementation of induced hypothermia in hospitals: existing guidelines promoting hypothermia are not sufficiently specific to be easily implemented, and practical impediments exist to the efficient implementation of induced hypothermia in busy Emergency Departments (EDs) and intensive care units (ICUs). To overcome these factors, we developed a knowledge translation program focused on the in-hospital care of patients that survive OHCA by disseminating a standardized treatment protocol, educational sessions, reminders, and audit-feedback to increase the use of therapeutic hypothermia in all eligible patients. The hypothesis of this large-scale study is that an effective and collaborative knowledge translation strategy for the 2005 AHA guideline on therapeutic hypothermia will result in an increase in post-cardiac arrest patients receiving appropriate therapeutic hypothermia.

## Methods/design

### The setting

The hospitals in this project include the 33 southern Ontario hospitals already participating in the University of Toronto regional coordinating centre site of the Resuscitation Outcomes Consortium (ROC), as well as 4 community hospitals from the regions of York and Simcoe. ROC is an international research collaborative studying interventions that may improve survival from OHCA [16,17]. Because the EDs of these hospitals are already engaged in out-of-hospital resuscitation research, participation in the Strategies for Post-Arrest Care (SPARC) Network is a natural extension of this research to include in-hospital care and ICUs. These 37 hospitals provide care to a population of 8.8 million people who live within eight regions of the Province of Ontario, and have hospital bed capacities ranging from 19 to >600 and ICU bed capacities ranging from 4 to 42. This sample also includes all of the adult teaching hospitals affiliated with McMaster University and the University of Toronto.

### Population

As part of the knowledge translation strategy, we will intentionally focus on using messaging that will simplify the decision about which patients to cool by using more liberal patient inclusion and exclusion criteria than is typically seen in the randomized trials of therapeutic hypothermia. All patients greater than 13 years of age, who have suffered a non-traumatic cardiac arrest, have a sustained return of spontaneous circulation (palpable pulse for >20 mins) and a Glasgow Coma Scale score less than 10 will be considered eligible to be cooled. Patients with a known 'do not resuscitate' (DNR) status limiting life saving interventions or the need for aortic balloon pump and/or cardiogenic shock will not be considered eligible for therapeutic hypothermia for the purposes of this trial.

### Sample size

During initial planning, there were insufficient data to perform a formal sample size calculation. Since the initial planning, we now have data from the one year retrospective collection from the participating hospitals that permit an approximation of study power. During recent years (2006 to 2008) the emergency medical services (EMS) in these regions transported approximately 1300 adults with OHCA to these destination hospitals. In 2008, 339 of these patients survived to be admitted to 30 of these hospitals, but only 10% were cooled to a body temperature less than 34 degrees Celsius within six hours of hospital arrival (intercluster correlation coefficient 0.09 estimated using variance components described by Hussey and Hughes [18]). The in-hospital mortality rate after successful resuscitation from OHCA is approximately 65% (range: 33% to 80% by institution). Assuming that a similar number of patients will be admitted to 37 hospitals in the network during two years of study, we anticipate that our study will have power (two-tailed Type I error probability 0.05) of at least 90% to detect an absolute increase of 1% or more in the proportion of patients that are successfully cooled to below 34°C within six hours of hospital arrival.

### Study design

A stepped wedge cluster randomized trial design will be used to evaluate the impact of this intervention [18]. With this design, the intervention will be implemented sequentially to the participating hospitals over a number of equally spaced time periods. The order in which the participating hospitals receive the intervention (*i.e.*, enter the 'active' phase) will be determined at random and, by the end of the random allocation, all hospitals will have received the intervention. The study intervention is applied at the level of the hospitals (clusters), making it impossible to randomize individual clinicians using a traditional randomized controlled trial. We decided to use the stepped wedge design for randomizing these clusters because we anticipate that the study intervention will do more good than harm (making a parallel design cluster randomized trial, in which certain hospitals do not receive the intervention unethical). The stepped wedge design is also appealing because of the large scope and size of our study; for practical reasons, it would be difficult to deliver the intervention simultaneously to all hospitals. Finally, a stepped wedge design offers a number of opportunities for data analysis, particularly for modeling the effect of time on the effectiveness of an intervention [18]. The wedge randomization will be computer-generated by the statistician on the project (KT).

**Figure 2:**
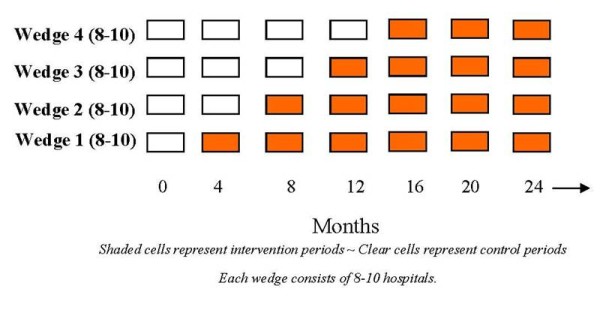
**Visual representation of the stepped wedge design used in this trial**.

The unit of analysis in this study will be patients adjusted for clustering within individual hospitals. Hospitals participating in the SPARC Network will be randomized in groups of 6-8 and stratified according to ICU size (<10 beds versus ≥10 beds) and participation in another recent large-scale quality improvement project [19]. The phases of the trial and the stepped wedge design are depicted in Figure 2.

### Intervention

To plan our intervention, we will first define the local barriers to implementation. These local barriers will be evaluated using a qualitative approach in a sample of the participating hospitals. We will conduct semi-structured interviews with various clinical staff from all participating hospitals and use a thematic analysis to determine the common barriers from the provider perspective. Conceptually, we anticipate that there will be several important barriers to the implementation of evidence-based care [20]: knowledge, *i.e.*, lack of understanding of how to implement guidelines promoting the use of induced hypothermia in patients after OHCA; attitudes, *i.e.*, clinicians' low expectations regarding clinical outcomes for survivors of OHCA; and behaviour, *i.e.*, barriers that interfere with clinicians successfully instituting induced hypothermia, for example insufficient resources, equipment, clinician time, and collaboration. The methods and results for this piece of the study will be published separately.

Our intervention will then be implemented during two phases: a 'passive phase' and an 'active phase' (Table 1). During the passive phase, we will conduct a site visit and provide a didactic presentation to participating hospitals' ED and ICU to introduce the SPARC collaborative goals and objectives, and to outline the rationale behind therapeutic hypothermia. The passive phase will occur according to the stepped wedge timeline, and marks the start of study participation but does not constitute the active intervention.

**Table 1:** Passive and active intervention phases

**Phase 1 - Passive**	• Identification of ED and ICU nurse and physician champion • Introductory site visit • Provide copy of standard hypothermia protocol
**Phase 2 - Active** **(intervention phase)**	• Site visit #2 with nurse facilitator • Presentation with staff • Cardiac arrest notification emails to ED and ICU champions • Monthly audit and feedback reports • Active implementation support • Invitation to videoconference education sessions

The active phase will commence approximately four months after the passive phase, also according to the stepped wedge timeline. The focus of this phase will be on using the information from the qualitative evaluation to create customized intervention tools and education for frontline staff in EDs and ICUs on when, how, and why to induce hypothermia and to increase its early use to improve the outcomes for post-cardiac arrest patients. To accomplish these objectives, we will focus on using strategies that will simplify the decision to cool patients and make it easier to carry out -- such as standardized protocols, visual reminders, and collaborative education about appropriate cooling methods in various situations. Specifically, this intervention will include:

1. Building a Collaborative Network: Access to the collaborative network of peer hospitals (the SPARC Network) that can share resources and experiences for the purpose of learning and improving (website, annual meetings, newsletters, blogs, *et al*.)

2. Reminders and Protocols: A standardized, evidence-based therapeutic hypothermia protocol and order set, developed by participant consensus and posted on the website for all hospitals. Innovative tools to help translate the guideline to the bedside and increase the use of therapeutic hypothermia in all eligible patients ('cooling kits,' checklists, reminders, stickers for cold intravenous fluids, and defibrillators). Email notifications to the site champions of patient transferred by EMS their site to enable and encourage follow-up on all cardiac arrest patients regarding decision making around cooling.

3. Education: Access to a multi-disciplinary educational program regarding post-resuscitation care with a focus on the use of therapeutic hypothermia in all eligible patients (lunch and learns, quarterly webinars, expert speaker sessions, video teleconferences).

4. Audit-Feedback: Access to real-time feedback on institutional practice including outcomes based on an integrated, web-based data collection system already in place.

### Data collection

Primary data collection will occur on all patients who are transferred to a participating hospital following an OHCA. Trained in-hospital data collectors will complete chart abstraction of the variables related to post-arrest care for each of the hospitals. The data dictionary, containing all variables, definitions, and ranges specific to the study and abstraction instructions will be programmed into a touch icon that displays the information at the point of data entry for each variable to ensure standardization. Training prior to data collection will be completed through web-based seminars with ongoing training via email reminders and web conferences. A web-based data collection tool will be employed to facilitate data collection across the geographical regions.

The in-hospital data will also be linked to an existing local OHCA registry [21]. This registry currently captures complete pre-hospital data, including the Utstein variables for uniform reporting of adult and paediatric cardiac arrests [22]. This local data set includes patient demographics and survival status to hospital discharge on every OHCA brought to the 37 participating hospitals. A random sample of 10% of abstracted patient charts across all data guardians will be periodically re-abstracted by centralized research staff for quality assurance purposes. All data will be anonymized and handled according to national privacy legislation and its related regulations.

### Outcomes and analysis

The primary outcome will be the proportion of OHCA patients that achieve the target temperature within six hours of ED arrival. Secondary outcomes will include the following: proportion of eligible patients where cooling was initiated anywhere within the hospitals; proportion of eligible patients where cooling was initiated within six hours of ED arrival; proportion of eligible patients where cooling was initiated (ever) in the ED; survival to discharge; neurological outcomes at discharge (modified Rankin Score [23], Cerebral Performance Category Scale [24]); mean and median time to target temperature; mean and median temperature at six hours from first ED arrival; and mean and median (inter-quartile range) of duration of cooling.

We will also evaluate unintended consequences of our intervention; for example, proportion of ineligible patients cooled, and proportion of patients cooled with contraindications for cooling.

The primary analysis will be to compare hospitals receiving the active intervention to those receiving the passive intervention according to the stepped wedge schedule, and adjusting for clustering within hospitals and temporal trends. We will conduct sensitivity analyses where all the comparisons are of active hospitals versus passive hospitals versus retrospective hospitals (*i.e.*, before study implementation), and adjusting for clustering within hospitals and temporal trends. Other sensitivity analyses will compare hospitals receiving any knowledge translation intervention (*i.e.*, active and passive) versus hospitals without the intervention (*i.e.*, retrospective data collection prior to any knowledge translation intervention).

Additionally, all study outcomes and their relationship to factors that influence whether or not there is appropriate uptake of the study intervention to improve care of patients after OHCA will be evaluated. For example, we will examine the effects of organizational and system factors that might lead to an imbalance between wedges, or to differential rates of uptake of our intervention. These organizational and system factors could include (but are not limited to): academic versus community (affiliation with a university); urban versus rural (bed size); cardiac arrest volume high versus low; intensivist versus non-intensivist staffing; participation in the ICU Clinical Best Practices Demonstration Project [19]; capability to perform percutaneous coronary interventions within the hospital; method of cooling used by the hospital; and rate of withdrawal of life support within the hospital. For all primary and most secondary analyses, we will use generalized estimating equations (GEE) to adjust for the effects of clustering.

### Research Ethics

This study has received individual Research Ethics Board approval from all 37 of the participating hospital sites.

## Discussion

This project is designed to translate knowledge into action [25] at the frontlines of healthcare using a collaborative network and a sustainable knowledge translation framework to help improve the care of patients who survive OHCA. We believe that this study, if successful, will improve patient outcomes and also help inform the design of system-wide quality improvement initiatives that target the care of these patients.

There have been few studies examining the effectiveness of system-wide interventions to improve the care of patients after OHCA. Herlitz *et al. *[26] showed that the adjusted one-month mortality of OHCA patients transported to hospital varied markedly (58% to 86%) due to differences in the level of post-resuscitation treatment provision at hospitals. In one of the few implementation studies conducted in post-resuscitation care, Sunde *et al. *[27] demonstrated that following implementation of a standardized post-resuscitation treatment protocol including the use of therapeutic hypothermia amongst other critical care interventions, the in-hospital survival, neurological outcome and one-year survival all markedly improved compared to historical controls. However, this study was limited by its inability to control for secular trends over time and was limited by its before-after design. We believe our proposed study has methodological strengths compared to previous research, and will help to advance science in post-resuscitation care. More broadly, it will provide information about the effectiveness of active versus passive versus no interventions applied across a diverse healthcare system and multiple hospitals.

We anticipate several challenges to conducting this study. First, clinician engagement is a frequently identified barrier in knowledge translation research. We will address this challenge by engaging all levels of clinical staff from the start of the project in a consensus-driven approach and by developing customized implementation tools. Second, timely and accurate data collection for a large-scale pragmatic study is also challenging, especially within busy EDs and ICUs. We will partner with an established and successful data collection system to ensure that comprehensive data collection is feasible in all participating hospitals. Third, our ability to influence healthcare workers across EDs, ICUs, and cardiology services may be challenged by institutional and specialty-specific cultural issues. We hope to overcome this limitation by recruiting local champions from all three of these disciplines to help ensure that this project is fully implemented at each site.

We believe our study intervention will lead to improved patient outcomes, and also provide a model for organizing system-wide quality improvement initiatives. The SPARC Network has the potential to become a collaborative network of hospitals that improves all aspects of post-resuscitation care. If it is successful, we anticipate that promotion and adoption of induced hypothermia will only represent the first step in an ongoing process to advance science in the fields of knowledge translation, quality improvement, and resuscitation science.

## Competing interests

Dr. Morrison is the Robert and Dorothy Pitts Chair in Acute Care and Emergency Medicine, Keenan Research Centre, Li Ka Shing Knowledge Institute, St Michael's Hospital, Past Chair of the Advance Cardiac Life Support Committee of the American Heart Association and the Co Chair of the Advance Life Support Task Force of the International Liaison Committee on Resuscitation for Consensus 2010. She is the Principal Investigator for the SPARC grant which was awarded peer reviewed funding from Heart and Stroke Foundation of Canada, CIHR and the Laerdal Medical Foundation. The remaining authors list no competing interests.

## Authors' contributions

KND helped conceive of the study and was directly involved in the design & implementation of the intervention and drafted the protocol manuscript. LJM conceived of the study, participated in its design, and helped to draft the protocol manuscript. DCS, PD, SB, GR, RW, NF, KT and DN were directly involved in the design and analytic plan for the study and edited the protocol manuscript. MZ developed the stepped wedge study design and edited the protocol manuscript. All authors read and approved the final manuscript.
